# Glutathione Peroxidase 3 as a Biomarker of Recurrence after Lung Cancer Surgery

**DOI:** 10.3390/jcm9123801

**Published:** 2020-11-24

**Authors:** Bo Gun Kho, Ha-Young Park, Hyun-Joo Cho, Cheol Kyu Park, Young-Chul Kim, Ju-Sik Yun, Sang-Yun Song, Kook-Joo Na, Yoo-Duk Choi, Seung-Won Lee, In-Jae Oh

**Affiliations:** 1 Lung and Esophageal Cancer Clinic, Chonnam National University Hwasun Hospital, 322 Seoyang-ro, Hwasun-eup, Hwasun-gun, Jeollanam-do 58128, Korea; imdrkbg@gmail.com (B.G.K.); ulabula77@naver.com (H.-Y.P.); repair2799@daum.net (H.-J.C.); ckpark214@jnu.ac.kr (C.K.P.); kyc0923@jnu.ac.kr (Y.-C.K.); jusikyun@gmail.com (J.-S.Y.); sysong@jnu.ac.kr (S.-Y.S.); kjna1125@hanmail.net (K.-J.N.); drydchoi@hanmail.net (Y.-D.C.); 2 Department of Internal Medicine, Chonnam National University Medical School, 160 Baekseo-ro, Dong-gu, Gwangju 61469, Korea; 3 Department of Thoracic and Cardiovascular Surgery, Chonnam National University Medical School, 160 Baekseo-ro, Dong-gu, Gwangju 61469, Korea; 4 Department of Pathology, Chonnam National University Medical School, 160 Baekseo-ro, Dong-gu, Gwangju 61469, Korea; 5 Department of Anatomy, Chonnam National University Medical School, 264 Seoyang-ro, Hwasun-eup, Hwasun-gun, Jeollanam-do 58128, Korea

**Keywords:** glutathione peroxidase 3, lung cancer, surgery, recurrence, biomarker

## Abstract

We aimed to examine the usefulness of serum glutathione peroxidase 3 (GPx3) as a biomarker of lung cancer recurrence after complete resection. We prospectively collected serial serum samples at the baseline, as well as 3, 6 and 12 months after surgery from complete resection cases in 2013. GPx3 levels were measured by enzyme-linked immunosorbent assay. Statistical tests including t-tests and Cox proportional hazard regression analyses were performed. Totally, 135 patients were enrolled, and 39 (28.9%) showed relapse during the median follow-up period (63.60 months; range, 0.167–81.867). The mean GPx3 change was significantly higher in the recurrence group at 6 months (0.32 ± 0.38 vs. 0.15 ± 0.29, *p* = 0.016) and 12 months (0.40 ± 0.37 vs. 0.13 ± 0.28, *p* = 0.001). The high GPx3 change group showed significantly higher 60-months recurrence rates than the low group (48.1% vs. 25.2% at 3 months, *p* = 0.005; 54.5% vs. 28.9% at 6 months, *p* = 0.018; 38.3% vs. 18.3% at 12 months, *p* = 0.035). High GPx3 change at 3 months were independent risk factors of recurrence (hazard ratio (HR) 3.318, 95% confidence interval (CI), 1.582–6.960, *p* = 0.002) and survival (HR 3.150, 95% CI, 1.301–7.628, *p* = 0.011). Therefore, serum GPx3 changes after surgery may be useful predictive biomarkers for recurrence in lung cancer. Larger-scale validation studies are warranted to confirm these findings.

## 1. Introduction

In 2018, lung cancer was the most frequently diagnosed cancer worldwide, and the disease is associated with the highest mortality values of all cancers [1,2,3]. Surgery is recommended for stage I and II lung cancer cases and some stage III cases. However, the recurrence rate is as high as 20% even in stage I disease [4,5], and this recurrence is associated with poor prognoses. Risk factors associated with lung cancer recurrence after surgery, in addition to the well-known TNM staging [5,6], include the degree of tumor differentiation [7,8,9], visceral pleural invasion [6,10,11], complete resection status [12,13], and angiolymphatic invasion [8,9]. However, serum protein biomarkers such as carcinoembryonic antigen, CYFRA 21-1 and neuron-specific enolase have not been investigated sufficiently in such settings, and do not show adequate sensitivity or specificity [14]. Therefore, there is a requirement for a study to identify a blood biomarker for the early detection of lung cancer recurrence.

The correlation between the development of cancer and reactive oxidative stress has been reported in several studies [15,16,17,18,19]. Reactive oxygen species (ROS) cause direct or indirect DNA damage, and are involved in the development of cancer through gene mutation and the alteration of signal transduction [15,16,17,18,19]. In addition, a correlation between ROS and angiogenesis and metastasis has been reported [18,19]. Antioxidant enzymes like nicotinamide adenine dinucleotide and glutathione peroxidase (GPx) provide resistance against oxidative stress development [19,20].

GPx3 is the only secretory form of the GPx family; it is a selenoprotein containing selenocysteine and acts as an antioxidant [21,22]. Hydrogen peroxide is detoxified through the oxidation of selenocysteine to selenic acid [23]. In this manner, GPx3 protects cells from oxidative stress [23]. The hypermethylation of GPx3 promotor CpG island induces the downregulation of GPx3 expression [24]. Serum GPx3 level downregulation has been observed in many cancers, while its upregulation is connected to the suppression of tumorigenesis [15,21,24,25,26]. Barret et al. found that the tumor number is increased in GPx3 knock out mice [21], suggesting that GPx3 has a role as a tumor suppressor and that its downregulation is related to tumor progression and proliferation.

A previous retrospective study reported the downregulation of GPx3 in lung cancer patients who underwent surgery [27]; therefore, serum GPx3 was proposed as a biomarker of early-stage lung cancer. Accordingly, we conducted this prospective study to examine the usefulness of serum GPx3 as a biomarker of recurrence after lung cancer surgery at a single institution.

## 2. Materials and Methods

### 2.1. Patients and Materials

A total of 165 patients underwent lung cancer surgery in Chonnam National University Hwasun Hospital in 2013. We defined ‘complete resection (CR)’ after discussions with thoracic surgeons as the satisfaction of all the following conditions: (1) having undergone segmentectomy or a more extensive range of operations (e.g., lobectomy or pneumonectomy), (2) sufficient lymph node dissection, or sampling, and (3) absence of postoperative stage IV disease. Exceptionally, we included 7 cases of wedge resection for ground-glass opacity nodules (GGNs) as CR cases, and their histology was invasive adenocarcinoma. Totally, 135 patients were classified into the CR group and enrolled for analysis. Serum samples were prospectively collected at the baseline, as well as 3, 6 and 12 months after surgery. All data were gathered in accordance with the amended Declaration of Helsinki, following the approval of the independent institutional review board (IRB) of Chonnam National University Hwasun Hospital (IRB approval number: CNUHH-2014-035).

Blood samples were collected in BD Vacutainer SS Plus Blood Collection Tubes (BD Biosciences, USA). For serum collection, samples were centrifuged using Rotina 380R centrifuge (Hettich, Germany) at 3000 rpm at 4 °C for 20 min. The samples were then stored at −180 °C until further laboratory use. GPx3 levels were measured three times per sample using the enzyme-linked immunosorbent assay (ELISA).

### 2.2. Statistical Analysis

For the statistical analysis, IBM^®^ SPSS^®^ Statistic version 25.0 was used. All ELISA data were expressed as mean ± standard deviation for continuous variables. When variables were normally distributed, the mean difference between the recurrence group and non-recurrence groups was tested using Student’s *t*-test or Welch’s *t*-test. To determine whether there existed a difference in the recurrence ratio between the variables, Pearson’s chi-square tests and Fisher’s exact tests were employed.

Kaplan–Meier analysis was performed using recurrence and recurrence time (months) as a status variable and time variable, respectively. Log-rank tests were used to test differences in the survival distributions across the subgroups of “GPx3 change”. The GPx3 change was defined as the ratio of the difference between the measured and baseline value and the baseline value (GPx3 change = (measured GPx3 − baseline GPx3)/baseline GPx3). The cutoff value in the prediction of recurrence was defined as the highest Youden index (sensitivity + specificity − 1), with sensitivity and specificity values of 70% or higher, based on receiver operating characteristic (ROC) curve analysis.

Cox proportional hazard regression analysis was performed to analyze the effect of GPx3 levels on survival using recurrence as a status variable and recurrence time as a time variable after controlling for confounding covariates such as smoking, age, histology, stage and adjuvant treatment. A *p*-value lower than 0.05 indicated statistical significance.

## 3. Results

### 3.1. Baseline Characteristics

Totally, 135 patients were assigned to the CR group, and underwent analysis. The median age was 63.48 ± 8.755 years: men (*n* = 81), 62.81 ± 8. 817 years; women (*n* = 54), 62.81 ± 8.701 years (Table 1). The proportion of smokers was 55.8% (current 28.5% and ex-smoker 27.3%). Histologically, adenocarcinoma was the most commonly observed cancer type (63.0%), followed by squamous cell carcinoma (25.9%). Postsurgical pathologic stage I disease accounted for 65.2% (stage IA 42.2% and IB 23.0%) of all such cases. The proportions of stage II and III disease were 21.5% and 12.6%, respectively. 

The most commonly employed surgery type was lobectomy (89.6%), while pneumonectomy was performed in 3.0% of the patients. Adjuvant treatments were performed in 41 (30.4%) patients, and comprised adjuvant chemotherapy (*n* = 35), postoperative radiation (*n* = 3), concurrent chemoradiation (*n* = 2), and chemotherapy followed by radiation (*n* = 1).

Thirty-nine (29.9%) patients were confirmed as having recurrence during the median follow-up period of 63.60 (range, 0.167–81.867) months. We divided them into two groups: the recurrence group and non-recurrence group.

### 3.2. Risk Factors for Recurrence

In order to identify the factors that may affect recurrence after surgery, we analyzed some known factors such as stage, pathologic invasion, and adjuvant treatment. Pathologic invasion was confirmed based on a pathologist’s report of visceral pleural invasion (*n* = 32), lymphovascular invasion (*n* = 22) or microscopic residual tumor on resection margin (*n* = 3). Because 7 patients had more than two factors, total 50 patients were classified into the group with pathological invasion. A chi-squared test and Fisher’s exact test were performed to determine the relationship between these variables and recurrence (Table 2).

In the groups without and with pathologic invasion, 17.6% (15/85) and 48.0% (24/50) of the patients, respectively, showed recurrence; the difference was statistically significantly different (*p* < 0.001, odds ratio = 4.308). On comparing the rates of recurrence in the stage I and non-stage I groups, 15.9% (14/88) and 53.2% (25/47) of those in the stage I and non-stage I (*p* < 0.001, odds ratio = 6.006) groups showed recurrence. In the group that did not receive adjuvant treatment, the lung cancer recurrence rate was 18.1% (17/94), while the corresponding value was 53.7% (22/41) among those in the other patient groups who received adjuvant treatment (*p* < 0.001, odds ratio = 5.245).

### 3.3. Mean GPx3 and GPx3 Change between the Two Groups

GPx3 values were measured at the baseline (before surgery), and 3 months, 6 months, and 12 months after surgery (Table 3). The mean GPx3 values at 3, 6, and 12 months after surgery did not show a statistically significant relationship with recurrence. The mean GPx3 level changes were significantly higher in the recurrence group at 6 months (0.32 ± 0.38 vs. 0.15 ± 0.29, *p* = 0.016) and 12 months (0.40 ± 0.37 vs. 0.13 ± 0.28, *p* = 0.001). 

### 3.4. Kaplan–Meier Curve Analysis of Recurrence and Survival

**Figure 1:**
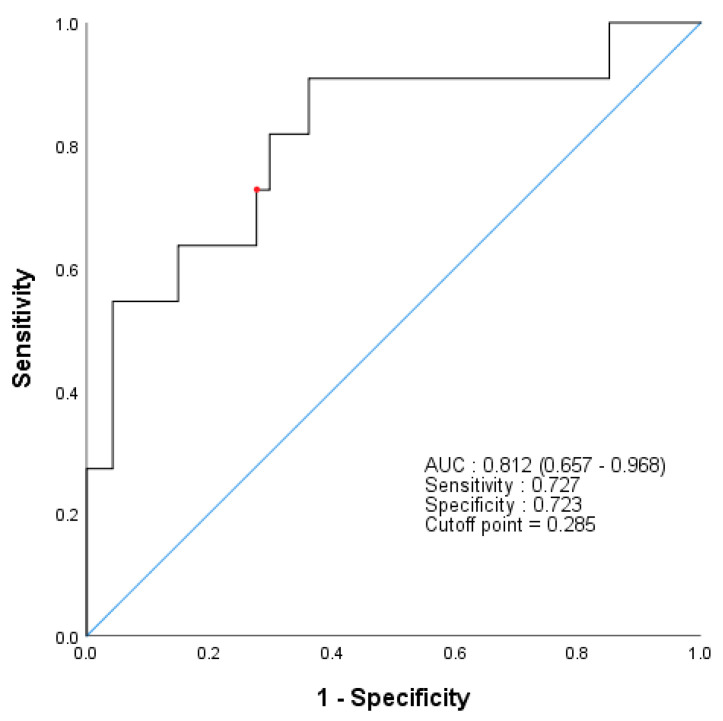
ROC curve of the GPx3 change in recurrence prediction. The AUC was 0.812 (95% CI, 0.657–0.986), and the cutoff point (0.285 μg/mL, red dot) was identified on the basis of the highest Youden index, with a sensitivity of 72.7% and specificity of 72.3%. Abbreviations: ROC, receiver operating characteristic; GPx3, glutathione peroxidase 3; AUC, area under the curve; CI, confidence interval.

**Figure 2:**
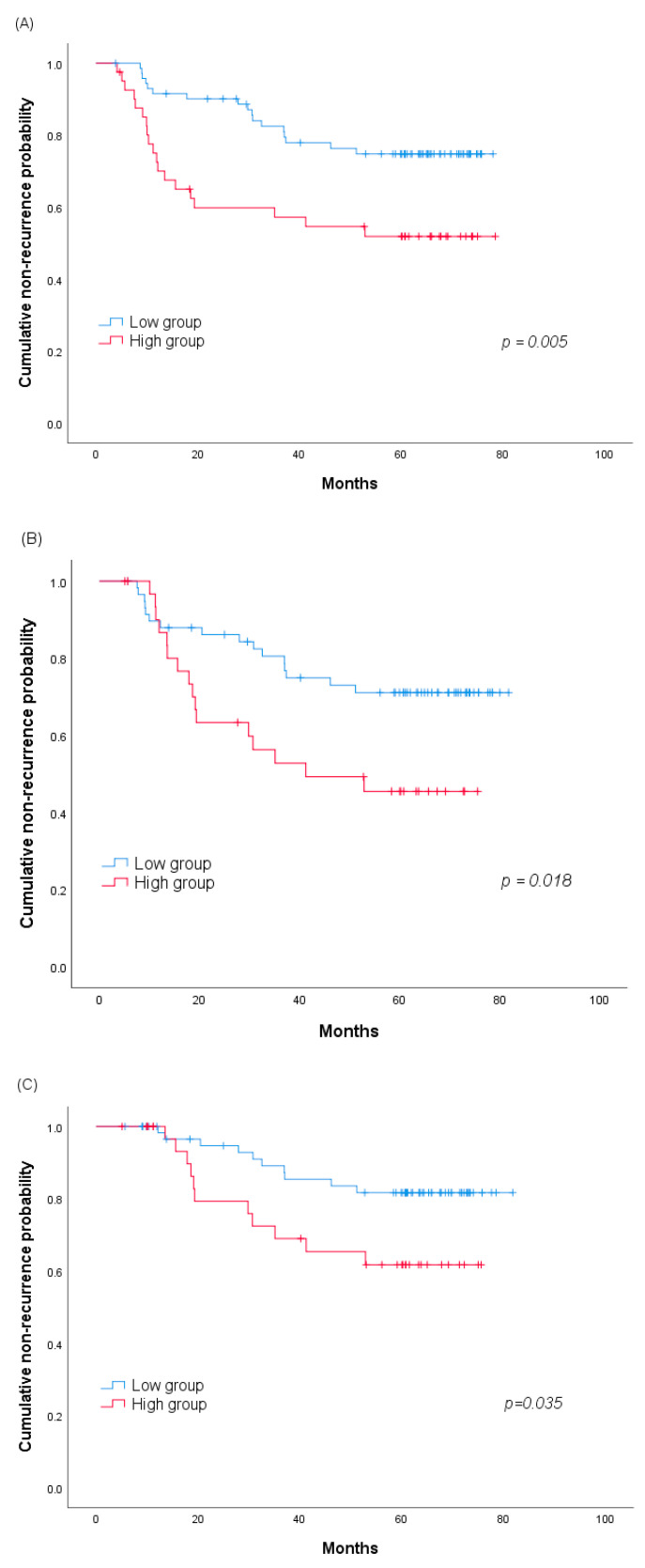
Kaplan–Meier analysis of the 60-months cumulative non-recurrence probability between the high and low groups according to the serum GPx3 change values (**A**) at 3, (**B**) 6 and (**C**) 12 months. Abbreviation: GPx3, glutathione peroxidase 3.

We hypothesized that the GPx3 change and recurrence rate would be related. To investigate this, we analyzed the GPx3 change and recurrence using Kaplan–Meier curves. Figure 1 shows the ROC curve on the basis of the GPx3 change for the prediction of recurrence in the group without pathologic invasion (*n* = 85). The area under curve for recurrence was 0.812 (95% confidence interval (CI], 0.657–0.968), and the cutoff value (0.285 μg/mL) was identified on the basis of the highest Youden index, with a sensitivity of 72.7% and specificity of 72.3%. The sensitivity, specificity, and Youden index in the prediction of recurrence for different cutoff values are shown in Table S1. We divided patients into the high and low groups based on the cutoff value, and the recurrence-free time after each measurement point was compared by a log-rank test. Lung cancer recurrence before each measurement was treated to censored data. At 3, 6 and 12 months, in terms of the GPx3 change, the high group showed a shorter time to recurrence, and all these differences were statistically significant (60-months recurrence rate 48.1% vs. 25.2% at 3 months, *p* = 0.005; 54.5% vs. 28.9% at 6 months, *p* = 0.018; 38.3% vs. 18.3% at 12 months, *p* = 0.035, Figure 2).

**Figure 3:**
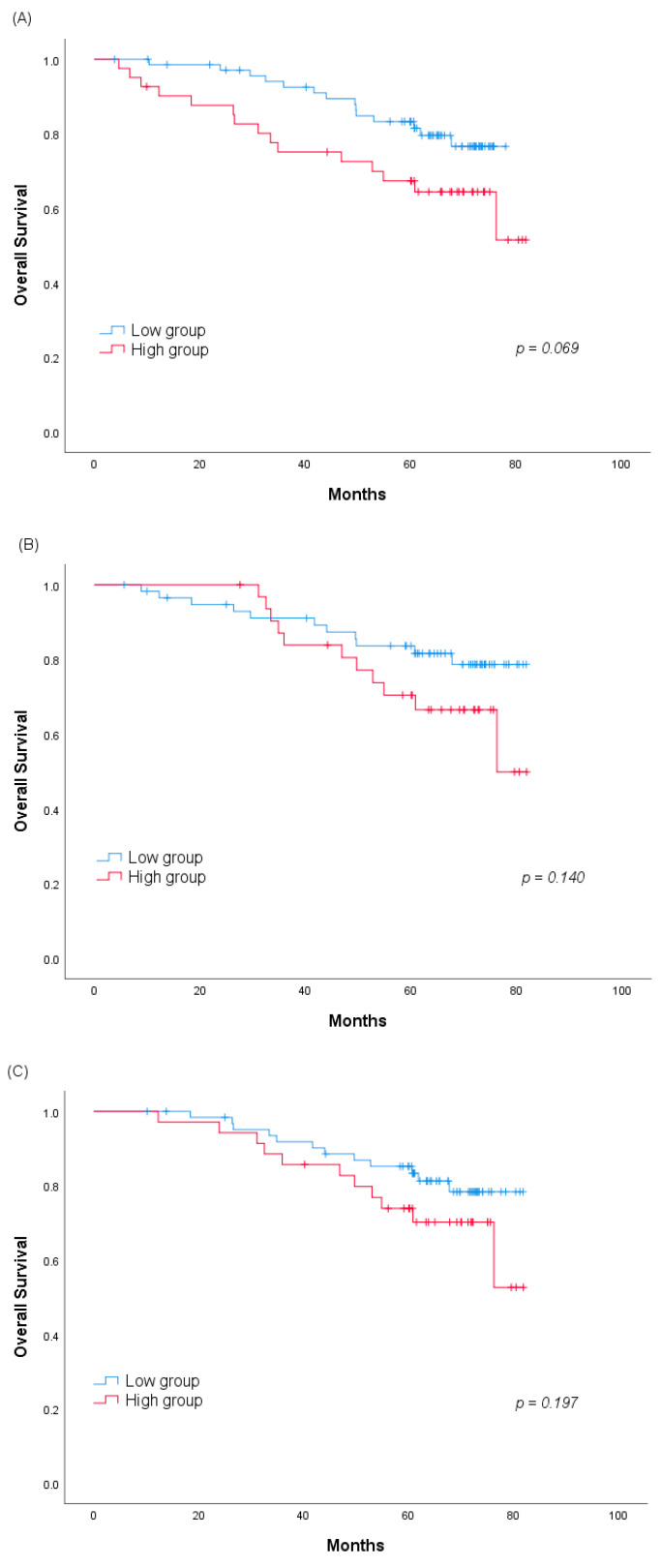
Difference in the 60-months overall survival between the high and low groups according to the serum GPx3 change (**A**) at 3, (**B**) 6 and (**C**) 12 months. Abbreviation: GPx3, glutathione peroxidase 3.

The effect of the cutoff value on the survival rate of patients was also assessed using the Kaplan–Meier curve. The high group tended to have lower survival rates than the low group at all the measurement time points (60-months survival rate 67.4% vs. 83.3% at 3 months, *p* = 0.069; 70.5% vs. 83.7% at 6 months, *p* = 0.140; 73.9% vs. 85.2% at 12 months, *p* = 0.197, Figure 3).

### 3.5. Multivariate Cox Regression of the GPx3 Change

To assess the impact of GPx3 on postoperative recurrence, we performed multivariate Cox regression analyses. Independent variables included age, sex, smoking, histology, stage, adjuvant treatment, pathologic invasion, and the GPx3 change at each month. Lung cancer recurrence before each measurement had been treated to censored data.

In all the measurements, the presence of a high GPx3 change (over cutoff value) showed statistical significance as a risk factor for recurrence. Except in cases of death or recurrence or those that were censored before the measurement point, high GPx3 changes were independent factors for postoperative recurrence at 3 and 12 months, but not 6 months (hazard ratio (HR) 3.318, 95% CI, 1.582–6.960, *p* = 0.002 at 3 months; HR 2.086, 95% CI, 0.907–4.795, *p* = 0.083 at 6 months; HR 4.018, 95% CI, 1.365–11.828, *p* = 0.012 at 12 months, Table 4). Additionally, pathologic invasion was an independent risk factor for recurrence at all the measurement time points. Disease stage was an independent risk factor at 3 and 6 months, but not 12 months. 

Using the same variables, we also investigated the hazard risk for death. In the Cox regression analysis, GPx3 change showed potential as a risk factor for death; it showed statistical significance for death at 3 and 6 months, but not 12 months (HR 3.150, 95% CI, 1.301–7.628, *p* = 0.011 at 3 months; HR 3.322, 95% CI, 1.055–10.462, *p* = 0.040 at 6 months; HR 2.435, 95% CI, 0.918–6.457, *p* = 0.074 at 12 months, Table 5). Adjuvant treatment and stage were also independent risk factors for death at all times points, and pathologic invasion showed a statistically significant effect at 3 and 12 months.

## 4. Discussion

In this study, we found that serum GPx3 changes after surgery may be useful predictive biomarkers for recurrence. We assigned patients to the experimental group if complete surgical resection of the lung cancer was achieved. The high GPx3 change group, at 3, 6 and 12 months postoperatively, showed significant associations with shorter recurrence-free durations than the low group. However, GPx3 change were not associated with overall survival by Kaplan–Meier analysis. In the Cox-regression analysis, values higher than the cutoff GPx3 change at 3 months were revealed as independent risk factors of recurrence and survival.

In the EAGLE study published in 2015, surgery was superior to non-surgical treatment in terms of survival in stage I-IIIA disease [5]. Lung cancer, including small cell lung cancer, is associated with a recurrence rate of 33.9% in stage I patients and 62.8% in stage IIIA patients who received surgery. The 1-year mortality in stage I patients before relapse was 2.7%, but increased to 48.3% after recurrence [5]. For non-small cell carcinoma (NSCLC), the recurrence rate in stage I disease after surgery was approximately 20% [4,28,29]. In stage I patients, the mean duration to postoperative recurrence was observed to be about 13 months [4]. In our study, the recurrence rates were 21.6% in stage I, 44.8% in stage II, and 80% in stage IIIA disease. Therefore, there is a need for more aggressive adjuvant treatments following the identification of populations with a recurrence risk using effective biomarkers.

We performed analyses among patients in whom it was suspected that the lung cancer was removed completely. CR is known to be associated with recurrence after surgery. In 2005, a definition was proposed by the Complete Resection Subcommittee, which is a subgroup of the International Association for the Study of Lung Cancer [13]; however, no clear consensus has been reached yet [12]. However, it seems clear whether CR is an important prognostic factor [12,30]. In cases with GGN, it is controversial whether wedge resection or segmentectomy yields better CR results [31]; however, several studies have reported the absence of significant differences between the two surgical methods in GGN [32]. Wedge resection of GGN was considered to lead to CR achievement in our analysis.

The risk factors for recurrence after the surgical resection of lung cancer have been reported [8,33]. Several studies have reported tumor size, visceral pleural invasion, angiolymphatic invasion, tumor grade, and complete surgical resection as significant risk factors of recurrence [33]. The TNM stage is widely accepted as a risk factor for recurrence and survival [8]. In our study, similarly, there was a statistically significant difference in the postoperative recurrence rates between the stage I cases and others. Several studies have reported that pathologic variables such as visceral pleural invasion and lymphovascular invasion are associated with cancer recurrence after surgery [8,10,11]. Visceral pleural invasion became an independent factor in determining the T stage regardless of tumor size since the publication of the TNM stage 7th edition [8,34]. Lymphovascular invasion is known to be associated with prognoses in various cancers [8]. In NSCLC, lymphovascular invasion has been accepted to be associated with relapse [9,10], but this is not yet reflected in the TNM staging. When the presence of visceral pleural invasion, lymphovascular invasion or microscopical residual tumor on resection margin was confirmed on histology, we defined the cases as having pathologic invasion that was also correlated to recurrence.

Interestingly, when multivariate Cox analysis was performed with several risk factors known to affect recurrence, the GPx3 change above the cutoff value (0.285 μg/mL) was found to be an independent risk factor for recurrence. In addition, the high GPx3 change at 3 months was revealed as significant factor related to survival.

GPx is an enzyme that reduces the levels of hydroperoxides, phospholipid hydroperoxides, and fatty acid hydroperoxide [24]. A total of eight sub-types constitute the GPx family; among them, GPx1 to GPx4 and GPx6 are selenoproteins that are found in humans [35]. GPx3 does not have an endoplasmic reticulum retention signal and is secreted into the plasma uniquely [23]. The expression of GPx3 has been observed in various organs including the liver, kidney, heart, lung and intestine [21,36]. The downregulation of GPx3 has been observed in numerous cancers including hepatocellular carcinoma [26], prostate cancer [37], gastric cancer [38], Barrett’s adenocarcinoma [39], glioblastoma [40], and cervical cancer [35]. Therefore, the potential of GPx3 as a tumor marker has been widely suggested. In vivo and in vitro studies have shown that GPx3 is associated with invasion, metastasis, recurrence, sensitivity to chemotherapy, prognoses, and tumorigenesis [24,26,35,37,39]. It is not clear how GPx3 aids in tumor suppression. In addition to protecting cells from oxidative stress for the prevention of tumorigenesis [19], one theory suggests that degree of tumorigenesis and metastasis is suppressed through intracellular signaling [41]. Recently, An et al. demonstrated, for the first time, that GPx3 suppresses the proliferation of lung cancer cells by the modulation of redox-mediated signals [42]. The study revealed that GPx3 prohibits the destruction of MAP kinase3 by ROS, finally suppressing cyclin B1 expression through the ErK/NF-kB pathway and arresting the cell cycle at the G2/M phase.

The main advantage of this study is the performance of serial prospective monitoring of serum GPx3 changes after surgical resection for the identification of a biomarker for recurrence. Most previous studies reported on gene expression using tissue samples [35,37,38] or plasma/serum GPx3 levels at specific time points such as the diagnostic phase [26,40]. However, this study has several limitations. First, it had a small sample size and a single-institution design. As a result, the absolute GPx3 value (not GPx3 change) was not associated with tumor recurrence; furthermore, we could not do separate analyses for several different pathological factors, such as pleural and lymphovascular invasion. Second, the validated cohort was not homogenous in terms of the pathologic stage, with a majority of the patients showing stage I lung cancer, and adjuvant therapy. To minimize the effect of heterogeneity, we used Cox proportional hazard model including other known risk factors such as adjuvant therapy, pathologic invasion and stage. However, the generalizability of our findings to cases with many other disease stages may be low. Third, the causal relationship between GPx3 and recurrence is not clear. Although it is unclear how the increase of a supposedly protective marker leads to an increase in recurrence, we suggest one explanation based on the review of Chang et al. [43]. GPx3 expression is decreased in patients with smoking-induced chronic obstructive pulmonary disease due to the chronic adaptations [44]. Conversely, GPx3 levels in the epithelial lining fluid of cigarette smokers is higher than non-smokers, probably in response to the increased exogenous ROS [45]. Similarly, there is a possibility that the GPx3 levels have elevated to resist the increased oxidative stress caused by recurrence of lung cancer patient during postoperative period. Fourth, this study did not reveal the origin of GPx3 production after complete resection. GPx3 upregulation can be also found in non-cancerous fibrotic lung tissues, such as those with idiopathic pulmonary fibrosis, a disease associated with oxidative stress [46]. 

In conclusion, the high GPx3 change group showed significantly higher recurrence rates than the low group. Additionally, a high GPx3 change at 3 months was revealed as an independent risk factor for recurrence and survival. Therefore, serum GPx3 change after surgery may be a useful predictive biomarker for recurrence in lung cancer. Further studies are warranted, to examine how GPx3 affects oxidant scavenging and redox signaling in the extracellular tumor microenvironment. 

## Figures and Tables

**Table 1:** Baseline demographics (N = 135).

Characteristics		No. (%)	Non-Recurrence	Recurrence	*p* Value
			(*n* = 96)	(*n* = 39)	
Sex	Female	54 (40)	39 (40.6)	15 (38.5)	0.816
	Male	81 (60)	57 (59.4)	24 (61.5)	
Age (y)		63.48 ± 8.76	63.42 ± 8.47	63.64 ± 9.54	0.893
	Female	62.81 ± 8.70	62.51 ± 8.91	63.60 ± 8.373	
	Male	63.9 ± 8.82	64.04 ± 8.06	63.67 ± 10.37	
Smoking history					0.491
	Never smoker	62 (45.9%)	44 (45.8)	18 (46.2)	
	Current smoker	39 (28.9%)	24 (25.0)	15 (38.5)	
	Ex-smoker	34 (25.2%)	28 (29.2)	6 (15.4)	
Histology					0.085
	ADC	85 (63.0)	61 (63.5)	24 (61.5)	
	SQC	35 (25.9)	23 (24.0)	12 (30.8)	
	LCC	7 (5.2)	7 (7.3)	0 (0.0)	
	SCLC	3 (2.2)	3 (3.1)	0 (0.0)	
	Mixed *	3 (2.2)	2 (2.1)	1 (2.6)	
	Others **	2 (1.5)	0 (0.0)	2 (5.1)	
Pathologic stage					<0.001
	ⅠA	57 (42.2)	48 (52.7)	9 (23.1)	
	ⅠB	31 (23.0)	26 (25.5)	5 (12.8)	
	ⅡA	25 (18.5)	15 (13.6)	10 (25.6)	
	ⅡB	4 (3.0)	2 (1.8)	2 (5.1)	
	ⅢA	17 (12.6)	4 (3.6)	13 (33.3)	
	ⅢB	1 (0.7)	1 (1.8)	0 (0.0)	
Surgery type					0.345
	Lobectomy	121 (89.6)	86 (89.6)	35 (89.7)	
	Pneumonectomy	4 (3.0)	2 (2.1)	2 (5.1)	
	Wedge	8 (5.9)	7 (7.3)	1 (2.6)	
	Wedge + lobectomy	1 (0.7)	1 (1.0)	0 (0.0)	
	Segmentectomy	1 (0.7)	0 (0.0)	1(2.6)	
Adjuvant treatment					<0.001
	None	94 (69.6)	77 (80.2)	17 (43.6)	
	Chemotherapy	35(25.9)	18 (18.8)	17 (43.6)	
	Chemo + Radiation	1 (0.7)	0 (0.0)	1 (2.6)	
	CCRT	2 (1.5)	0 (0.0)	2 (5.1)	
	Radiation	3 (2.2)	1 (1.0)	2 (5.1)	

Values are presented as number (%) or median with standard error. * Mixed: small cell lung carcinoma with adenocarcinoma (*n* = 2), adenosquamous cell carcinoma (*n* = 2), large cell neuroendocrine cell carcinoma with adenocarcinoma (*n* = 1). ** Others: pleomorphic carcinoma (*n* = 2), adenoid cystic carcinoma (*n* = 1), mucoepidermoid carcinoma (*n* = 1). Abbreviations: ADC, adenocarcinoma; SQC, squamous cell carcinoma; LCC, large cell carcinoma; SCLC, small cell lung cancer; CCRT, concurrent chemoradiation.

**Table 2:** Risk factors for recurrence.

Risk Factors		Non-Recurrence	Recurrence	Total	*p* Value	Odds Ratio
Pathologic invasion	Absent	70 (82.4%)	15 (17.6%)	85 (100%)	<0.001	4.308
	Exist	26 (52.0%)	24 (48.0%)	50 (100%)		
Stage	Stage I	74 (84.1%)	14 (15.9%)	88 (100%)	<0.001	6.006
	Others	22 (46.8%)	25 (53.2%)	47 (100%)		
Adjuvant therapy	No	77 (81.9%)	17 (18.1%)	94 (100%)	<0.001	5.245
	Yes	19 (46.3%)	22 (53.7%)	41 (100%)		

**Table 3:** Mean GPx3 and GPx3 change between the recurrence and non-recurrence groups.

Variables (μg/mL)	Non-Recurrence	Recurrence	*p* Value
GPx3 at baseline (*n* = 135)	6.30 ± 1.76 (*n* = 96)	5.89 ± 1.50 (*n* = 39)	0.20
GPx3 at 3 months (*n* = 113)	7.90 ± 3.50 (*n* = 77)	7.93 ± 2.37 (*n* = 36)	0.96
GPx3 at 6 months (*n* = 114)	6.79 ± 1.82 (*n* = 57)	7.46 ± 2.26 (*n* = 34)	0.13
GPx3 at 12 months (*n* = 119)	7.17 ± 2.09 (*n* = 65)	7.97 ± 2.10 (*n* = 34)	0.08
GPx3 change at 3 months	0.22 ± 0.40	0.35 ± 0.35	0.115
GPx3 change at 6 months	0.15 ± 0.29	0.32 ± 0.38	0.016
GPx3 change at 12 months	0.13 ± 0.28	0.40 ± 0.37	0.001

Values are presented as number (%) or mean with standard deviation. GPx3 change = (measured GPx3 − baseline GPx3)/baseline GPx3. Abbreviation: GPx3, glutathione peroxidase 3.

**Table 4:** Multivariate Cox regression for mean GPx3 change effect on recurrence.

Time Points	Variables	Subgroups	HR (95% CI)	*p* Value
3 months	Adjuvant therapy	No vs. Yes	0.565 (0.196–1.632)	0.291
	Pathologic invasion	Absent vs. exist	5.301 (2.124–11.917)	<0.001
	Stage	Stage I vs. others	6.103 (2.110–17.653)	0.001
	GPx3 change at 3 months	<0.285 vs. >0.285	3.318 (1.582–6.960)	0.002
6 months	Adjuvant therapy	No vs. Yes	0.590 (0.166–2.102)	0.416
	Pathologic invasion	Absent vs. exist	3.326 (1.331–8.311)	0.010
	Stage	Stage I vs. others	4.907 (1.359–17.711)	0.015
	GPx3 change at 6 months	<0.285 vs. >0.285	2.086 (0.907–4.795)	0.083
12 months	Adjuvant therapy	No vs. Yes	0.330 (0.048–2.292)	0.262
	Pathologic invasion	Absent vs. exist	6.787 (1.787–25.770)	0.005
	Stage	Stage I vs. others	6.046 (0.922–39.628)	0.061
	GPx3 change at 12 months	<0.285 vs. >0.285	4.018 (1.365–11.828)	0.012

Abbreviations: GPx3, glutathione peroxidase 3; HR, hazard ratio; CI, confidence interval.

**Table 5:** Multivariate Cox regression for mean GPx3 change effect on death.

Time Points	Variables	Subgroups	HR (95% CI)	*p* Value
3 months	Adjuvant therapy	No vs. Yes	0.189 (0.051–0.692)	0.012
	Pathologic invasion	Absent vs. exist	3.084 (1.260–7.551)	0.014
	Stage	Stage I vs. others	8.611 (2.457–30.181)	0.001
	GPx3 change at 3 months	<0.285 vs. >0.285	3.150 (1.301–7.628)	0.011
6 months	Adjuvant therapy	No vs. Yes	0.148 (0.031–0.693)	0.015
	Pathologic invasion	Absent vs. exist	2.175 (0.719–6.582)	0.169
	Stage	Stage I vs. others	16.457 (3.813–71.039)	<0.001
	GPx3 change at 6 months	<0.285 vs. >0.285	3.322 (1.055–10.462)	0.040
12 months	Adjuvant therapy	No vs. Yes	0.133 (0.026–0.693)	0.017
	Pathologic invasion	Absent vs. exist	3.405 (1.176–9.861)	0.024
	Stage	Stage I vs. others	10.241 (2.310–45.410)	0.002
	GPx3 change at 12 months	<0.285 vs. >0.285	2.435 (0.918–6.457)	0.074

Abbreviations: GPx3, glutathione peroxidase 3; HR, hazard ratio; CI, confidence interval.

## Supplementary Materials

The following is available online at https://www.mdpi.com/2077-0383/9/12/3801/s1: Table S1: Sensitivity and specificity in the prediction of postoperative recurrence on the basis of different cutoff GPx3 change values.
