# Supplementary material for: Glutathione Peroxidase 3 as a Biomarker of Recurrence after Lung Cancer Surgery
Source: J Clin Med. 2020 Nov 24;9(12):3801. doi: 10.3390/jcm9123801 (PMC7760369; doi:10.3390/jcm9123801)
Supplement: Supplementary file 1 [file jcm-09-03801-s001.pdf]

Supplementary Table 1. Sensitivity and specificity in predicting postoperative recurrence on the basis of different cutoff values of the GPx3 change.

**Value**	**Sensitivity**	**Specificity**	**Youden index**	**Euclidean distance**
1.007	0.273	1.000	0.273	0.727
0.813	0.273	0.979	0.251	0.728
0.511	0.545	0.957	0.503	0.457
0.446	0.545	0.915	0.460	0.462
0.371	0.545	0.851	0.397	0.478
0.285	0.727	0.723	0.451	0.388
0.261	0.727	0.702	0.429	0.404
0.251	0.818	0.702	0.520	0.349
0.230	0.818	0.681	0.499	0.367
0.209	0.818	0.660	0.478	0.386
0.190	0.909	0.638	0.547	0.373
0.187	0.909	0.617	0.526	0.394
0.180	0.909	0.596	0.505	0.414
0.099	0.909	0.447	0.356	0.561

*Youden index = [Sensitivity + Specificity − 1]
